# F8 Inversions at Xq28 Causing Hemophilia A Are Associated With Specific Methylation Changes: Implication for Molecular Epigenetic Diagnosis

**DOI:** 10.3389/fgene.2019.00508

**Published:** 2019-05-29

**Authors:** Muhammad Ahmer Jamil, Amit Sharma, Nicole Nuesgen, Behnaz Pezeshkpoor, André Heimbach, Anne Pavlova, Johannes Oldenburg, Osman El-Maarri

**Affiliations:** ^1^Institute of Experimental Hematology and Transfusion Medicine, University of Bonn, Bonn, Germany; ^2^Institute of Human Genetics, School of Medicine, University of Bonn – University Hospital Bonn, Bonn, Germany

**Keywords:** structural rearrangement, epigenetic, DNA methylation, inversion, hemophilia, molecular diagnosis

## Abstract

Diverse DNA structural variations (SVs) in human cancers and several other diseases are well documented. For genomic inversions in particular, the disease causing mechanism may not be clear, especially if the inversion border does not cross a coding sequence. Understanding about the molecular processes of these inverted genomic sequences, in a mainly epigenetic context, may provide additional information regarding sequence-specific regulation of gene expression in human diseases. Herein, we study one such inversion hotspot at Xq28, which leads to the disruption of F8 gene and results in hemophilia A phenotype. To determine the epigenetic consequence of this rearrangement, we evaluated DNA methylation levels of 12 CpG rich regions with the coverage of 550 kb by using bisulfite-pyrosequencing and next-generation sequencing (NGS)-based bisulfite re-sequencing enrichment assay. Our results show that this inversion prone area harbors widespread methylation changes at the studied regions. However, only 5/12 regions showed significant methylation changes, specifically in case of intron 1 inversion (two regions), intron 22 inversion (two regions) and one common region in both inversions. Interestingly, these aberrant methylated regions were found to be overlapping with the inversion proximities. In addition, two CpG sites reached 100% sensitivity and specificity to discriminate wild type from intron 22 and intron 1 inversion samples. While we found age to be an influencing factor on methylation levels at some regions, covariate analysis still confirms the differential methylation induced by inversion, regardless of age. The hemophilia A methylation inversion “HAMI” assay provides an advantage over conventional PCR-based methods, which may not detect novel rare genomic rearrangements. Taken together, we showed that genomic inversions in the F8 (Xq28) region are associated with detectable changes in methylation levels and can be used as an epigenetic diagnostic marker.

## Introduction

Implications of human DNA sequence variations have received considerable attention in recent years and structural variants (SVs) are considered an important contributor among them. SVs, particularly inversions, can vary in size from few nucleotides to large-scale chromosomal rearrangements. The inversions can have functional consequences by truncating a given gene (or genes) or by rearranging the regulatory element in the local proximity, both having a disproportionate impact on gene expression and transcriptional variability (Puig et al., 2015).

**FIGURE 1:**
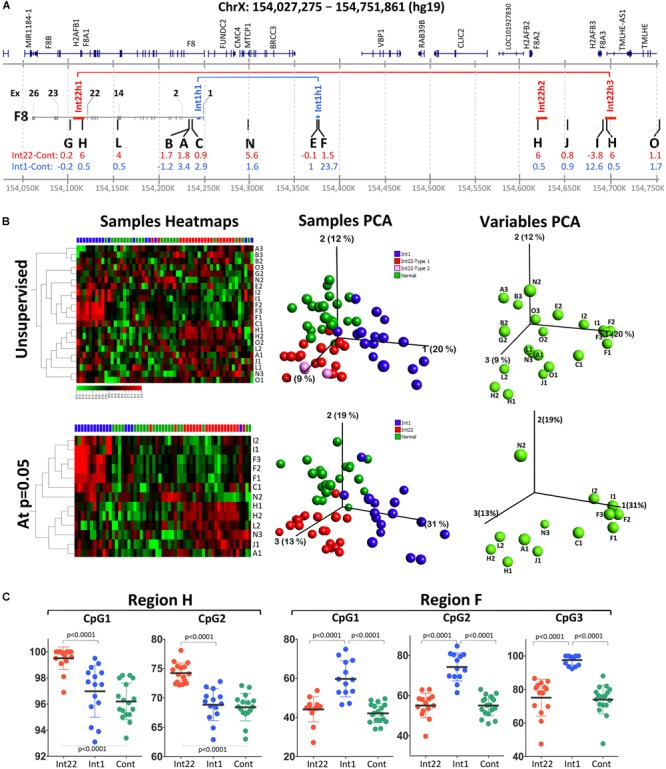
Pyrosequencing methylation data on 12 selected regions from intron 22 and intron 1-inversion samples as well as healthy male controls. **(A)** Detailed map on X chromosome (Chr X: 154,027,275-154,751,861:hg19) showing *F8*, the three Int22h and the two Int1h repeats involved in the inversion mutations. The positions of the studied regions are indicated in the middle region by capital letters. The inversion prone regions are labeled with red and blue horizontal lines for intron-22 and intron-1 inversions, respectively. **(B)** Methylation data represented by heatmaps, sample PCA and variable PCA plots. **(C)** Detailed data of the methylation values for individual samples at the two best regions (H and F) that clearly distinguish between inverted and non-inverted control samples.

Hemophilia A (OMIM #306700), an inherited bleeding disorder, harbors two such rearrangements at chromosome X (Xq28) involving the coagulation factor VIII (F8) gene. *F8* (∼186 kb; 26 exons) is located at the telomeric end of the X- chromosome and contains regions with high GC content, which makes it more susceptible toward the methylated cytosine deamination mutations [2,537 mutations, reported by CDC Hemophilia A Mutation Project (CHAMP) (Payne et al., 2013)]. In addition, two hotspot inversions (known as intron 1 and intron 22 inversions) are reported accounting for 40–50% of patients with severe hemophilia A (Andrikovics et al., 2003; Oldenburg and El-Maarri, 2006; Zimmermann et al., 2011). These hotspot recurring inversions are caused by intra-chromosomal homologous recombination between identical inverted repeats: two long repeats located within the *F8* locus: the Int22h-1 in intron 22 and the Int1h-1 in intron 1 (Lakich et al., 1993; Naylor et al., 1995). The former is 9.1 kb in length and has two additional homologs (Int22h-2 and 3) at about 500–580 kb distance toward the telomere, while the latter is about 1 kb and has one homolog located 141 kb toward the telomere (Figure 1) (UCSC genome browser). Both repeats are prone to intra-chromosomal homologous recombination leading to an inversion of the intervening sequence, thus leaving the *F8* split into two parts of opposite transcriptional direction (Bagnall et al., 2002, 2006). The clinical result of such inversions is a severe hemophilia A (HA) phenotype with no functional FVIII protein. Inversion events leading to human diseases are not limited to *F8* gene, other genes, such as *IDS* gene (Hunter syndrome), *MSH2* gene (Lynch syndrome), *EML4-ALK* rearrangement in non-small cell lung cancer (NSCLC), *AP3B1* (Hermansky-Pudlak syndrome type 2), have been previously implicated (Bondeson et al., 1995; Soda et al., 2007; Jones et al., 2013; Rhees et al., 2014).

However, the effect of a given DNA inversion may not be as clear as the above examples. It is not known whether SVs without a clear gene-destruction effect are still benign in nature. For instance, it is likely that an inversion can disturb normal chromatin architecture and this could be translated into interchanging of hetero- and euchromatic states. This would lead to abnormal methylation patterns, and ultimately to alterations in gene expression that may have a phenotypic impact. Thus, a given gene could cross the borders between an actively transcribed and a non-active region as a result of the inversion. A clear example is what has been observed in *Drosophila* position-effect variegation, where an inversion of DNA shifts the w+ and rst+ genes from an euchromatin to a heterochromatin domain, thus resulting in white color eyes (Schotta et al., 2003).

In humans, indications for the none gene breaking effects of inversions came from Gonzalez et al. (2014) who reported on the effect of a common 0.45 Mb inversion at 16p11.2 on local gene expression and found that inverted alleles strongly correlated to neighboring gene expression. Expression effects were seen on single copy genes within the inverted regions as well as on genes flanking the duplicated regions (where the inversion breakpoints occur). Additionally, the multiple copy genes located in the duplications were also affected. Some genes are over-expressed, while others are under-expressed in the inverted allele. However, a large proportion remained unaffected. Although the molecular mechanism behind this set of observations goes beyond the scope of this particular study, it provides an indication for a cause-effect relationship between common human inversions and gene expression and its link to a disease phenotype: the joint susceptibility to asthma and obesity.

To date, there is no molecular mechanism that explains the biogenesis for the inversion effect on expression. Furthermore, it is still not possible to predict the effects of a given inversion. It has been hypothesized that changes in the chromatin structure comprise a possible underlying reason, but a clear model detailing the interplay between a given methylation and the different parameters that affect the gene expression, such as histones modifications, DNA methylation, nucleosome occupancy and three-dimensional chromatin structure, remains elusive. The above-described *F8* inversions are well characterized and their breakpoints are within defined unique repeats regions. Therefore, these two inversions are a suitable model for investigating the effect of inversions on gene expression as well as chromatin structure and epigenetic modifications.

In this study, we took advantage of the *F8* gene inversions model to analyze DNA methylation levels of CpG rich regions within and flanking the inverted DNA regions in wild type and inverted DNA (with intron 1 or intron 22 inversions). In summary, our results show clear detectable DNA methylation changes associated with inversions that are flanking the inverted regions. Therefore, methylation aberrations are a useful diagnostic tool to identify inversion structural variations.

## Materials and Methods

### DNA Samples

DNA samples corresponding to healthy controls (21 non-hemophilic males) and to male hemophilia patients with known intron 1 (16 samples) or intron 22 inversions (19 samples) were obtained from the hemophilia center at the Institute of Experimental Hematology and Transfusion Medicine (University Clinic Bonn, Germany) and from the Institute for Human Genetics (University of Wuerzburg, Germany). The samples used are derived from DNA collected for molecular diagnostic purposes. All blood samples from patients and healthy controls were obtained upon written informed consent. The Ethics Committee of the University Clinic Bonn authorized the use of pre-collected DNA samples for research purposes (approval number 091/09 date 05/06/2009).

### Methylation Analysis and Pyrosequencing Assays

CpG rich regions within and around the inverted sequences were identified using the UCSC website. Feasible regions for primer designs were selected for methylation analysis. Primers for PCR amplifications as well as pyrosequencing primers were designed using the PyroMark assay-design Q24 software (Qiagen, Germany). Bisulfite treatment was done using the EZ 96-DNA methylation kit (Zymo Research, Irvine, CA, United States) following manufacturer’s protocol. Bisulfite PCRs were done using HOT FIREPol (Solis Biodyne, Tartu, Estonia). Pyrosequencing was done on a PyroMark Q24 or Q96 machine (Qiagen, Germany). Primers used for amplification are listed in Supplementary Table S1. In total, 12 different regions were studied and designated as regions A to O.

### Verification of Results by Bis-Seq NGS Based Assay

Since the pyrosequencing assay is restricted to check only few individual CpGs and provides an estimated average of methylation the results had to be verified by covering relatively larger regions and the spatial relationships (phase) between different CpGs in the same region had to be revealed. Such data could be provided by NGS-based resequencing assays. For this purpose, we chose the SeqCap Epi Enrichment system from NimbleGen (Roche, Switzerland). Using this system, we targeted the *F8* region: chrX: 154,027,275-154,751,861. Samples included four intron 1, six intron 22 inversions and four wild type controls. After obtaining the data we filtered for the overlapping reads with our pyrosequencing assays. All data are submitted to EBI as a mapped “BAM” file under study accession number “ERP113762.”

### Next Generation Sequencing Analysis

Sequencing data was generated using Illumina HiSeq 2500 v4 with read length of 2 × 125 bp. Reads were generated in fastq file format. Reads were pre-filtered for any adapters’ sequence. Reads quality was tested using fastqc^1^ and all reads were passed for the quality cut-off of 10. Reads were mapped using BSMAP (Xi and Li, 2009) program to HG38 genome downloaded from UCSC with parameter settings to WGBS mode (−*s* = 16), all four strands mapping (−*n* = 1) and with parallel computing of four processor cores (−*p* = 4). Mapped reads were split into top and bottom strand using bamtools (Barnett et al., 2011) to separately remove duplicates for both strands. Duplicates were removed using the “MarkDuplicates” function in picard tool^2^. Removed duplicate removed strands were merged together into single mapped file using bamtools. Filtered reads were filtered again for the properly paired reads using bamtools filter with parameters of “-isMapped true,” “isPaired true” and “isProperPair true.” Properly paired reads were further processed using “clipOverlap” function in “bamUtil” (Jun et al., 2015) to clip overlapping paired-end reads to correct bias for methylation calculation. Methylation percentages were determined using the “methRatio.py” function in BSMAP with the parameters of minimum number of reads per CpGs set to 1 (*m* = 1) and report to zero methylation (−z). A final methylation table with number of Cs, Ts and coverage for every CpG was created by removing the uncovered region via NimbleGen. Methylation analyses were further carried out in R using the “methylkit” (Akalin et al., 2012) package. Fisher’s exact test was performed to calculate the *p*-value between samples for every CpG site.

### Statistical Analysis and Data Visualization

Statistical analysis was done using R or Prism (GraphPad software). Additional data analysis and visualization were done using Qlucore Omics Explorer (Sweden) and ProFit software from Quatum Soft (Switzerland). Regression analyses using R were performed to understand the effect of covariates (age). Formula for regression analysis used were “aov(lm(MethDiff∼CaseControl+Age+CaseControl^∗^Age).”

## Results

### CpGs Regions at the Border of the Inverted DNA Are Prone to Significant Differential Methylation

The main aim of this study was to detect differential methylation region(s) that could serve as markers for identification of *F8* inversions rearrangements. We initially designed and selected the regions based on (1) feasibility of reading methylation of at least three CpGs, (2) their presence in a region between the three prime regions of F8 and the Int22h3 repeat regions, and (3) their presence in non-repetitive regions (like L1 and Alu). Next, we could retain 12 regions whose methylation was neither constantly 0 nor 100% for all samples: i.e., variable methylation. We then studied three groups of samples: int22 and int1 inversions and healthy controls. Three of the regions failed to show significant statistical differences when applying statistical test to compare between the groups, namely regions G, E, and O (Figure 1).

The rest of the nine regions showed statistical significance for at least one CpG at one of three comparisons (Figure 1 and Supplementary Table S2). For intron 22 inversion samples, eight individual CpGs in six regions (regions H, L, A, N, J, and I) were statistically different compared to healthy controls. The most significant region was region H (average meth. diff. = 6% at CpG2; *t*-test *p* < 0.0001) embedded within the Int22h repeats, followed by region L in exon 14 of factor 8 (average meth. diff. = 4% at CpG2; *t*-test *p* = 0.0005).

For intron 1 inversion samples, eight individual CpGs were statistically also significantly differentially methylated in comparison to healthy controls (Figure 1 and Supplementary Table S2), covering five different regions: A, C, F, J, and I. The most significant region was region F embedded in the Int1h repeat (average meth. diff. = 23.7% at CpG3; *t*-test *p* < 0.0001), followed by region C (average meth. diff. = 2.9% at CpG1; *t*-test *p* = 0.0004). However, region I showed higher average differential methylation reaching 12.6%, but a *p*-value of 0.0011.

Of significance, the regions that showed the highest differential methylation were situated either within the repeats involved in the homologous recombination leading to the inversion (region H: intron 22 inversion and region F: intron 1 inversion) or close to that border (region C, L, and I).

### Two Regions Show Promising Biomarkers Properties: High Sensitivity and Specificity, Making Them Eligible as Diagnostic Markers

In order to use methylation at a given CpG as a diagnostic marker to detect inverted DNA we calculated sensitivity and specificity for each CpG that showed a statistical significance difference between inversions and healthy controls. For this purpose, we defined sensitivity as the fraction of the inverted DNA sample that is identified as differentially methylated in comparison to the wild type controls. Whereas specificity is defined as the fraction of healthy samples within the normal range of methylation and not overlapping with inverted DNA. For intron 1 and intron 22 inversions, a sensitivity and specificity of 1 were reached for region F CpG3 and region H CpG2 (Supplementary Table S2).

### Investigation of Factors Influencing DNA Methylation: Age and DNA Polymorphism

#### Age Effect: Healthy Group Shows Statistically Significant Linear Correlation Between Age and Methylation at Regions F and I and a Clear Tendency at Region L

A significant correlation between age and methylation difference was observed for some CpG sites. In order to understand whether the difference is due to age or rearrangements of the inversion region, we performed rigorous regression analysis between inversion samples and controls with age as a covariate (Supplementary Figure S2). Regression analysis revealed that some CpGs sites, i.e., F-CpG1, F-CpG2, F-CpG3, I-CpG1, and I-CpG2, showed statistical significance between intron-1 inversion and control in the difference between age and methylation and the difference between phenotype and methylation, while the difference between age and phenotype was not found to be significant. Thus, the difference in methylation due to intron-1 inversion will be statistically significant at any age range (Supplementary Figure S2A). Regarding intron-22 inversion, we found no statistically significant difference between age to methylation or phenotype to methylation (Supplementary Figure S2B).

In order to re-emphasize the age effect on region F and to exclude an effect on the ability to discriminate wild type from inversions at any age group, we calculated observed – expected – methylation levels for all samples in the healthy and the intron 1 inversion groups. For this purpose, we calculated the expected methylation values according to an equation of best fit linear regression model of healthy samples for each of the three CpGs and the average of the three CpGs in region F (Supplementary Figure S3A). Comparison of observed and expected levels showed a highly significant difference only at intron1 samples (at all three CpGs), which indicates that the observed differences between intron 1 inversions and healthy controls are not solely due to an age effect (Supplementary Figure S3B). Moreover, observed methylation values minus calculated expected methylation values (according to age using the linear regression fitting equation of the healthy samples) revealed high significance between healthy controls and intron 1 inversion compared to intron 22 inversions (Supplementary Figure S3C). This once more indicates that the differences in comparison to healthy controls are largely due to the intron 1 inversion of DNA.

### DNA Polymorphism Effect

As the DNA polymorphism may affect the level of methylation at neighboring CpGs, we searched the UCSC databases for occurrences of polymorphisms in a window of 1 Kb surrounding each investigated CpG. The results are displayed in Supplementary Table S3. While we found some SNPs with minimum allele frequency up to 0.21 in European populations, especially in the two regions with high discrimination power to detect intron 1 (region F) and intron 22 (region H), no reported SNPs with MAF > 0.05 have been reported. Therefore, we could largely exclude a broader effect of polymorphism on the level of methylation at the two relevant regions H and F.

### Methylation Correlation Between Different Regions Suggests Stochastic Random Effect, While Top Differentially Methylated Regions Are Indeed Correlated

**FIGURE 2:**
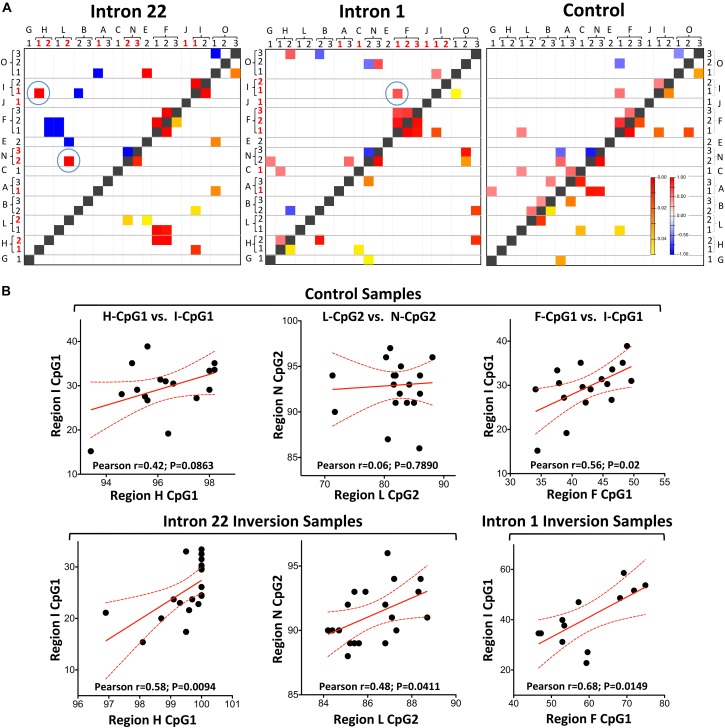
Correlation analysis between the studied CpG sites. **(A)** Heatmaps representing Pearson correlation (left upper triangle) and *p*-values (right lower triangle). The CpG rich region names are labeled with capital letters, while the individual CpGs are labeled with numbers, whereby red ones represent statistically significant ones. When two significant CpGs from two regions are correlated they are highlighted with a blue circle. **(B)** Correlation graphs of the circled ones of part A. The best fit linear curves as well as the 95% confidence intervals are shown in red.

In intron 1 and intron 22 inversions, we observed abnormal methylation at several CpG sites. Therefore, we queried whether these changes are coordinated and if they are correlated. In other words, are these changes in methylation in parallel at two or more altered regions for a given inversion type. If this is the case, a statistically significant correlation should be observed. Indeed, we calculated all correlations pairwise for all 22 CpG sites for every group (intron 22, intron 1 and healthy controls as separate groups). While we observed 15, 15, and 16 inter CpGs correlations in intron 22, intron 1 and healthy controls, we found little overlaps between all three groups. This was mainly observed at the intra-CpG correlations within the regions N and F (Figure 2A). The absence of overlap suggests a change in the nature of epigenetic marks from the normal non-inverted to an inverted DNA.

In this context, three inter region correlations were observed in inversion groups that involve regions that are differentially methylated between inversions and controls. Two of these are observed in intron 22 only and are not present in controls, namely H-CpG1 vs. I-CpG1 and L-CpG2 vs. N-CpG2 (Figure 2). Possibly, this is specific for the inversion samples and is induced by the rearrangement. This hypothesis is supported by two arguments: (1) all four involved CpGs are at the top differentially methylated between intron 22 inversions and controls and (2) such correlation is absent in normal samples.

Yet, the third correlation was observed in intron 1 samples between F-CpG1 and I-CpG1. These CpGs also showed a significant methylation difference between intron 1 inversion samples and controls. In fact, these two CpGs are the highest two differentially methylated CpGs with an average difference between intron 1 samples and controls of 23.7 and 12.6% for F-CpG1 and I-CpG1, respectively. However, this correlation is induced by the age effect on methylation as this involves two CpGs that show high correlation between age and methylation (Supplementary Figure S2). In addition, this correlation is observed in healthy controls, re-emphasizing that the correlation between age and methylation is the driving force behind this correlation between the CpG1 at region F and CpG1 at region I.

### Targeted Bisulfite Re-sequencing Largely Confirms the Pyrosequencing Results

#### Confirmation of Bisulfite Pyrosequencing

**FIGURE 3:**
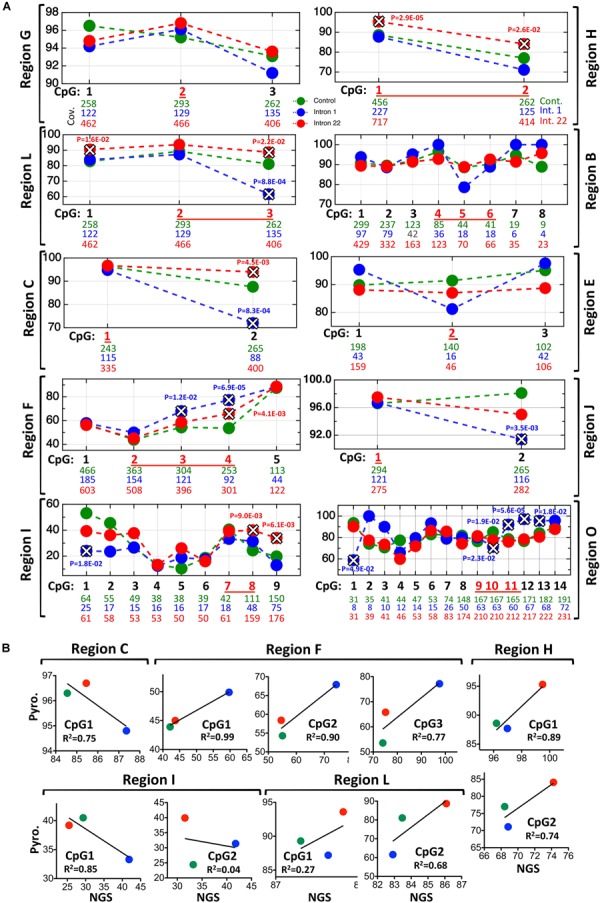
NGS results of the studied regions shown in Figure 1A. **(A)** Each graph represents one region; regions A and N have no enough coverage and are absent. The number of reads for each CpG is shown below the corresponding CpG, the *p*-value of Fisher’s exact test is shown when significant (marked by X) between healthy samples (green) and intron 1-inversion samples (blue) or intron 22-inversion samples (red). The corresponding pyrosequencing CpGs are in red and underlined. **(B)** Correlation graphs between the pyrosequencing and the NGS methylation levels results.

**FIGURE 4:**
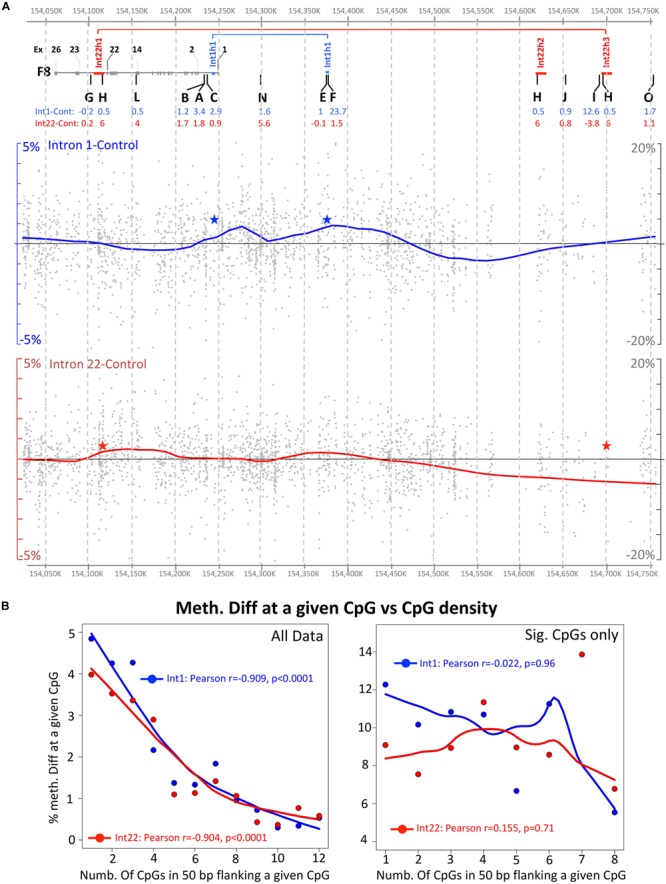
Global visualization of NGS data in the *F8* region (hg19: Chr X 154,027,275–154,751,861). **(A)** Upper panel shows the relative positions of the studied pyrosequencing regions, the middle panel shows the NGS data for intron 1 inversion samples and the lower panel the intron 22 inversion samples. The covered individual CpG methylation data are represented by a gray dot, while additionally, the data is represented by a smooth curve representing the trend of changes between the inverted and the control samples. CpG sites with less than 30 coverage or overlapping with known SNPs or repeats were excluded. Red and blue stars indicate the DNA inversion junctions. **(B)** Correlation between the methylation differences at a given CpG and the density of CpG within 50 bp flanking region. Left and right side include all CpG data and only significant data (Fisher’s exact test), respectively.

In order to further confirm the above results via an alternative method, we performed targeted bisulfite re-sequencing with the SeqCap Epi Enrichment system from NimbleGen (Roche, Switzerland) to capture a region containing the *F8* and extending up to the extragenic Int22h repeats (hg19, ChrX: 154,027,275–154,751,861; Figure 3, 4). Ten of the studied pyrosequencing regions could be covered, while two were insufficiently covered with low read counts (regions A and N). In order to increase coverage and to decrease the effect of inter-individual differences, we merged the reads that belonged to the same group of samples. This resulted in a pool of reads of three groups including six, two and four individual DNA samples for intron 22 inversion, intron 1 inversion and healthy controls, respectively. Moreover, this merging approach increased the read numbers at each CpG site with ranges 23-717, 4-227, and 9-466 for intron 22 inversion, intron-1 inversion and healthy controls, respectively.

Nineteen individual CpGs were overlapping between the pyrosequencing and the NGS enrichment approach, of which 12 were showing complete concordance in results of significance (Figure 3 and Supplementary Figure S1). This is also reflected by the correlation of average methylation between both approaches across the three samples cohorts (Figure 3B). Of particular interest are the two highly differentially methylated regions that showed high sensitivity and high specificity for distinguishing inversions from non-inversions, namely regions F and H for detecting intron 1 and intron 22, respectively. Both showed high significance in correlation values and overlapping, confirming results in both methods. The bisulfite targeted enrichment analysis provided additional confidence in the inversion-induced methylation aberrations and in the ability of such methylation assays to detect the inversions.

### Trend Line of Methylation Changes Over the *F8* Till Intr22h3 Covered Region

Next, a global trend line was drawn of the methylation differences including all CpGs captured by the enrichment protocol (i.e., not only overlaps with pyrosequencing results as presented in the previous section). For this approach, we filtered the data to exclude any CpG overlapping with a repeat or a known SNP. Additionally, we excluded data for any CpG that had less than 30 reads in one of the two compared categories. In a next step, a trend line of difference in methylation to the healthy male controls was plotted in a map showing relative position to the studied pyrosequencing regions (Figure 4A). As expected, this approach indicated a major hypermethylated domain overlapping with the regions F and H for intron 1 and intron 22, respectively. However, we also noticed that the inversion breakpoints (shown as blue and red stars in Figure 4A) are lying in “methylation-disturbed” domains. The largest methylation disturbance in both magnitude and length of the domain appear to be overlapping with the inversion junctions. All of the above suggest that the observed methylation alterations are indeed reflection of new genomic architecture caused by the DNA inversion.

### Characteristics of CpGs Showing Differential Methylation

We investigated the relationship between the degree of CpG methylation difference and the density of CpGs in a window of 50 bp where the CpG in question is in the center (Figure 4B). Using all data for all CpGs (regardless of statistical significance) we found that a clear and highly significant relationship between the methylation differences and CpG density exists where relatively CpG dense regions are more stable and show smaller methylation differences. This applies for the comparisons healthy vs. intron 1 (*r* = −0.909, Fisher’s exact test *p* < 0.0001) and healthy vs. intron 22 (*r* = −0.904, Fisher’s exact test *p* < 0.0001) (Figure 4B). It is our opinion that this is a general phenomenon of variability of methylation at “stand-alone” CpGs where they are more prone to uncontrolled “natural” fluctuations. However, the CpG methylation at significantly differentially methylated CpGs failed to show this correlation indicating that the latter are the result of induced aberrant methylation due to DNA rearrangement. From this analysis, we conclude that statistically significant methylation changes are more likely to occur at CpG rich regions or at clusters rather than at sole dispersed (non-clustered) CpGs.

## Discussion

The human genome shows significant variability between individuals (Auton et al., 2015). This variability is caused by single nucleotide polymorphisms, deletions, duplications, translocations and inversions. The effect of which may either be detectable as a change in the phenotype (which include disease manifestation) or be benign without observable phenotype. The molecular mechanism for the former can be explained for SNPs, deletions or duplications by virtue of possible changes in the DNA sequences leading to altered gene expression or protein structure. However, in the cases of translocations or inversions, there is no net gain or loss in DNA. Therefore, the association to a phenotype is difficult to explain by DNA changes unless the breakpoints disrupt a coding sequence or an expression-regulatory element (like a promoter or enhancer) (Harewood and Fraser, 2014). However, an additional scenario could be responsible to cause a phenotype: a shift in chromatin structure or – as it is also known – a position effect variegation (PEV).

Position effect variegation is one of similar phenomena which occur due to relocation of a genomic segment from one region to another and it has been extensively studied in *Drosophila*, yeast, mice and cultured human cells (Tham and Zakian, 2002; Pedram et al., 2006; Elgin and Reuter, 2013; Tchasovnikarova et al., 2015). Inversion prone position effects are not only limited to other species, it has also been reported in some human disease conditions, such as aniridia (*PAX6*), campomelic dysplasia (*SOX9*), familial adenomatous polyposis (*APC*) and Saethre-Chotzen syndrome (*TWIST1*) (Fantes et al., 1995; de Chadarevian et al., 2002; Cai et al., 2003; Velagaleti et al., 2005). Of note, some inversion variants can also act as risk factor for the offspring in microdeletion syndromes, such as Williams–Beuren syndrome, Angelman syndrome and Sotos syndrome (Osborne et al., 2001; Gimelli et al., 2003; Visser et al., 2005).

The above would lead to recreation of chromatin domains that result in local and regional epigenetic changes like DNA methylation aberrations. In this study, we used the two inversion hotspots in the *F8* gene at Xq28 as a model to investigate the global methylation aberration. Indeed, we found specific changes associated with each of the two inversions. With one specific region for each of the inversions showing high sensitivity and high specificity, our results pave the way for the use of methylation-based assay to detect the inversion. The hemophilia A methylation inversion “HAMI” assay will have several advantages over traditional assays. It is noteworthy to mention that repetitive elements also play an important role in generating structural variants (SVs) in humans (Xing et al., 2009). Among all mobile element types, long interspersed element-1 (LINE-1, or L1) has been previously investigated for DNA methylation-related changes in diseased conditions (Nusgen et al., 2015; Sharma et al., 2019). In this particular study, we took advantage of one such full length L1 repeat (region O) located in the vicinity of the *F8* gene and evaluated the methylation status of this repeat in both inversion type patients. However, no differences were found between inversion and wild type.

Currently, the gold standard molecular diagnostic assay to detect the inversion is the inverse based PCR assay (Rossetti et al., 2005), a procedure that needs up to 2–3 working days to complete and requires a skilled technician to perform a critical ligation step. In comparison, the HAMI assay includes three fail-free steps: (1) bisulfite conversion, (2) PCR and (3) quantitative pyrosequencing, all of which could be performed in 1 day. An additional advantage for HAMI is that it does not detect a specific DNA junction. Therefore, no specific amplification primers to detect only known inversions are required, while any rearrangement that could still be missed by specific amplification across known rearrangement junctions will be detected. However, disadvantages and limitations of such an assay include establishment of controls to define the relative borders (cut-off) of normal levels, as this could be population- or ethnicity-specific.

Overall, we could determine the methylation levels at multiple regions surrounding/overlapping *F8* associated genomic inversions at Xq28 region. Further evaluations are required to establish whether these epigenetic changes are cause or consequence of these inversion events.

## Data Availability

The datasets generated for this study can be found in the ENA, https://www.ebi.ac.uk/ena/data/view/PRJEB31235.

## Ethics Statement

The Ethic committee of the University Clinic in Bonn authorized the use of pre-collected DNA samples for research purposes, approval number 091/09 date 05/06/2009.

## Author Contributions

MJ, AS, and OE-M wrote the manuscript. MJ, AS, BP, and OE-M analyzed the results. MJ and OE-M performed the bioinformatics analysis. AS and NN performed the experiments. BP, AH, AP, and JO provided the samples and infrastructure and commented on the manuscript. OE-M designed the study.

## Conflict of Interest Statement

The authors declare that the research was conducted in the absence of any commercial or financial relationships that could be construed as a potential conflict of interest.

## Footnotes

1 http://www.bioinformatics.babraham.ac.uk/projects/fastqc

2 http://broadinstitute.github.io/picard/

## Supplementary Material

FIGURE S1 Detailed pyrosequencing methylation data on 12 selected regions from intron 22 and intron 1-inversion samples as well as healthy male controls. **(A)** Detailed map of X chromosome (154,027,275–154,751,861:hg19) showing the F8, the three Int22h and the two Int1h repeats involved in the inversion mutations. The positions of the studied regions are represented in the middle by capital letters, below which is the max-observed difference in methylation average. The inversion prone regions are labeled with red and blue horizontal lines for the intron-22 and intron-1 inversions, respectively.(B) Vertical scatter plots represent the detail methylation levels of individual studied samples for all individual CpG sites. The significant *t*-test is shown in the figure.

FIGURE S2 Age covariate regression analysis showing correlation between age and methylation levels for healthy controls in comparison to intron 1-inversion samples **(A)** and to intron 22-inversion samples. **(B)** Every plot shows methylation data vs. age. Above the individual plots are Pearson correlation p and rho values, while below the *p*-values of age-covariate analysis are shown. All significant *p*-values are written in bold. In case of significant Pearson correlation, the values are labeled with solid transparent red, green or blue rectangles. The plots corresponding to significant differences between cases and controls, even after considering age as covariates (*P*-value. MethDiff∼CaseControls), are indicated by a red cadre.

FIGURE S3 Calculation of observed vs. expected methylations values according to predicted linear regression formula of methylation vs. age of healthy group. **(A)** Linear regression curves of methylation vs. age for the three groups of samples (intron 1-inversion samples, intron 22-inversion samples and healthy controls). Also, equations are shown for every CpG (red, green, and blue for CpGs 1, 2, and 3, respectively) and for the average methylation of three CpGs (in black). **(B)** Comparison between observed and calculated expected values according to the linear regression equation of healthy controls. *T*-test *p*-values showed significance of all CpGs and their average only in the intron 1 inversion group. **(C)** Comparisons of observed-expected methylation values between inversion groups and healthy controls.

TABLE S1 Primers list used in this study.

TABLE S2 Summary values of data presented in Figure 1 together with calculated sensitivity and specificity.

TABLE S3 Common SNPs around the studied CpG site.
