# Supplementary material for: F8 Inversions at Xq28 Causing Hemophilia A Are Associated With Specific Methylation Changes: Implication for Molecular Epigenetic Diagnosis
Source: Front Genet. 2019 May 29;10:508. doi: 10.3389/fgene.2019.00508 (PMC6548806; doi:10.3389/fgene.2019.00508)
Supplement: FIGURE S2 — Age covariate regression analysis showing correlation between age and methylation levels for healthy controls in comparison to intron 1-inversion samples (A) and to intron 22-inversion samples. (B) Every plot shows methylation data vs. age. Above the individual plots are Pearson correlation p and rho values, while below the p-values of age-covariate analysis are shown. All significant p-values are written in bold. In case of significant Pearson correlation, the values are labeled with solid transparent red, green or blue rectangles. The plots corresponding to significant differences between cases and controls, even after considering age as covariates (P-value. MethDiff∼CaseControls), are indicated by a red cadre. [file Data_Sheet_1.PDF]

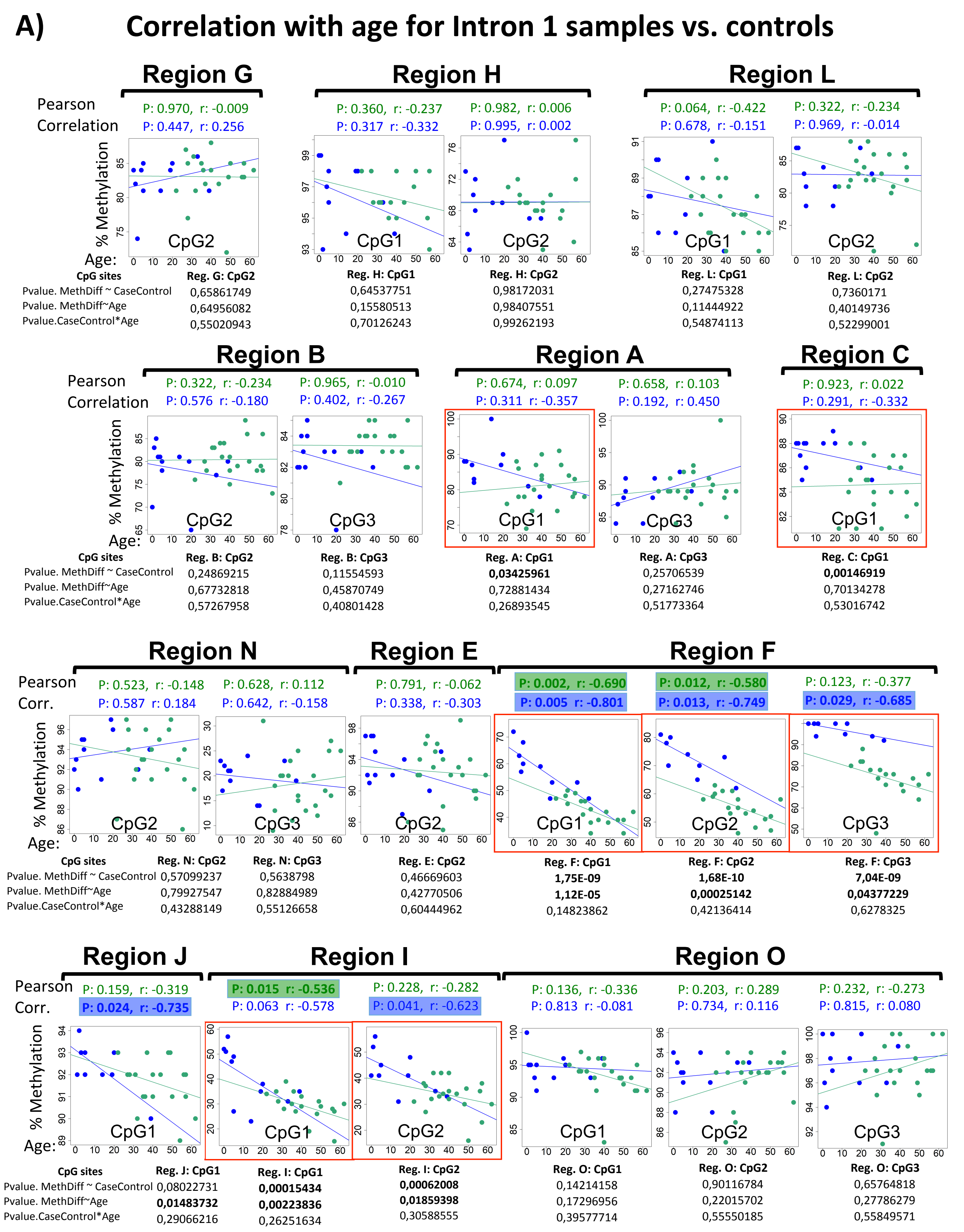

A) Correlation with age for Intron 1 samples vs. controls
Region G
Region H
Region L
Pearson Correlation
P: 0.970, r: -0.009
P: 0.447, r: 0.256
P: 0.360, r: -0.237
P: 0.317 r: -0.332
P: 0.982, r: 0.006
P: 0.995, r: 0.002
P: 0.064, r: -0.422
P: 0.678, r: -0.151
P: 0.322, r: -0.234
P: 0.969, r: -0.014
% Methylation
CpG2
CpG1
CpG2
CpG1
CpG2
Age:
CpG sites
Reg. G: CpG2
Reg. H: CpG1
Reg. H: CpG2
Reg. L: CpG1
Reg. L: CpG2
Pvalue. MethDiff ~ CaseControl
0,65861749
0,64537751
0,98172031
0,27475328
0,7360171
Pvalue. MethDiff~Age
0,64956082
0,15580513
0,98407551
0,11444922
0,40149736
Pvalue.CaseControl*Age
0,55020943
0,70126243
0,99262193
0,54874113
0,52299001
Region B
Region A
Region C
Pearson Correlation
P: 0.322, r: -0.234
P: 0.576 r: -0.180
P: 0.965, r: -0.010
P: 0.402, r: -0.267
P: 0.674, r: 0.097
P: 0.311 r: -0.357
P: 0.658, r: 0.103
P: 0.192, r: 0.450
P: 0.923, r: 0.022
P: 0.291, r: -0.332
% Methylation
CpG2
CpG3
CpG1
CpG3
CpG1
Age:
CpG sites
Reg. B: CpG2
Reg. B: CpG3
Reg. A: CpG1
Reg. A: CpG3
Reg. C: CpG1
Pvalue. MethDiff ~ CaseControl
0,24869215
0,11554593
0,03425961
0,25706539
0,00146919
Pvalue. MethDiff~Age
0,67732818
0,45870749
0,72881434
0,27162746
0,70134278
Pvalue.CaseControl*Age
0,57267958
0,40801428
0,26893545
0,51773364
0,53016742
Region N
Region E
Region F
Pearson Corr.
P: 0.523, r: -0.148
P: 0.587 r: 0.184
P: 0.628, r: 0.112
P: 0.642, r: -0.158
P: 0.791, r: -0.062
P: 0.338, r: -0.303
P: 0.002, r: -0.690
P: 0.005 r: -0.801
P: 0.012, r: -0.580
P: 0.013, r: -0.749
P: 0.123, r: -0.377
P: 0.029, r: -0.685
% Methylation
CpG2
CpG3
CpG2
CpG1
CpG2
CpG3
Age:
CpG sites
Reg. N: CpG2
Reg. N: CpG3
Reg. E: CpG2
Reg. F: CpG1
Reg. F: CpG2
Reg. F: CpG3
Pvalue. MethDiff ~ CaseControl
0,57099237
0,5638798
0,46669603
1,75E-09
1,68E-10
7,04E-09
Pvalue. MethDiff~Age
0,79927547
0,82884989
0,42770506
1,12E-05
0,00025142
0,04377229
Pvalue.CaseControl*Age
0,43288149
0,55126658
0,60444962
0,14823862
0,42136414
0,6278325
Region J
Region I
Region O
Pearson Corr.
P: 0.159, r: -0.319
P: 0.024, r: -0.735
P: 0.015 r: -0.536
P: 0.063 r: -0.578
P: 0.228, r: -0.282
P: 0.041, r: -0.623
P: 0.136, r: -0.336
P: 0.813 r: -0.081
P: 0.203, r: 0.289
P: 0.734, r: 0.116
P: 0.232, r: -0.273
P: 0.815, r: 0.080
% Methylation
CpG1
CpG1
CpG2
CpG1
CpG2
CpG3
Age:
CpG sites
Reg. J: CpG1
Reg. I: CpG1
Reg. I: CpG2
Reg. O: CpG1
Reg. O: CpG2
Reg. O: CpG3
Pvalue. MethDiff ~ CaseControl
0,08022731
0,00015434
0,00062008
0,14214158
0,90116784
0,65764818
Pvalue. MethDiff~Age
0,01483732
0,00223836
0,01859399
0,17296956
0,22015702
0,27786279
Pvalue.CaseControl*Age
0,29066216
0,26251634
0,30588555
0,39577714
0,55550185
0,55849571

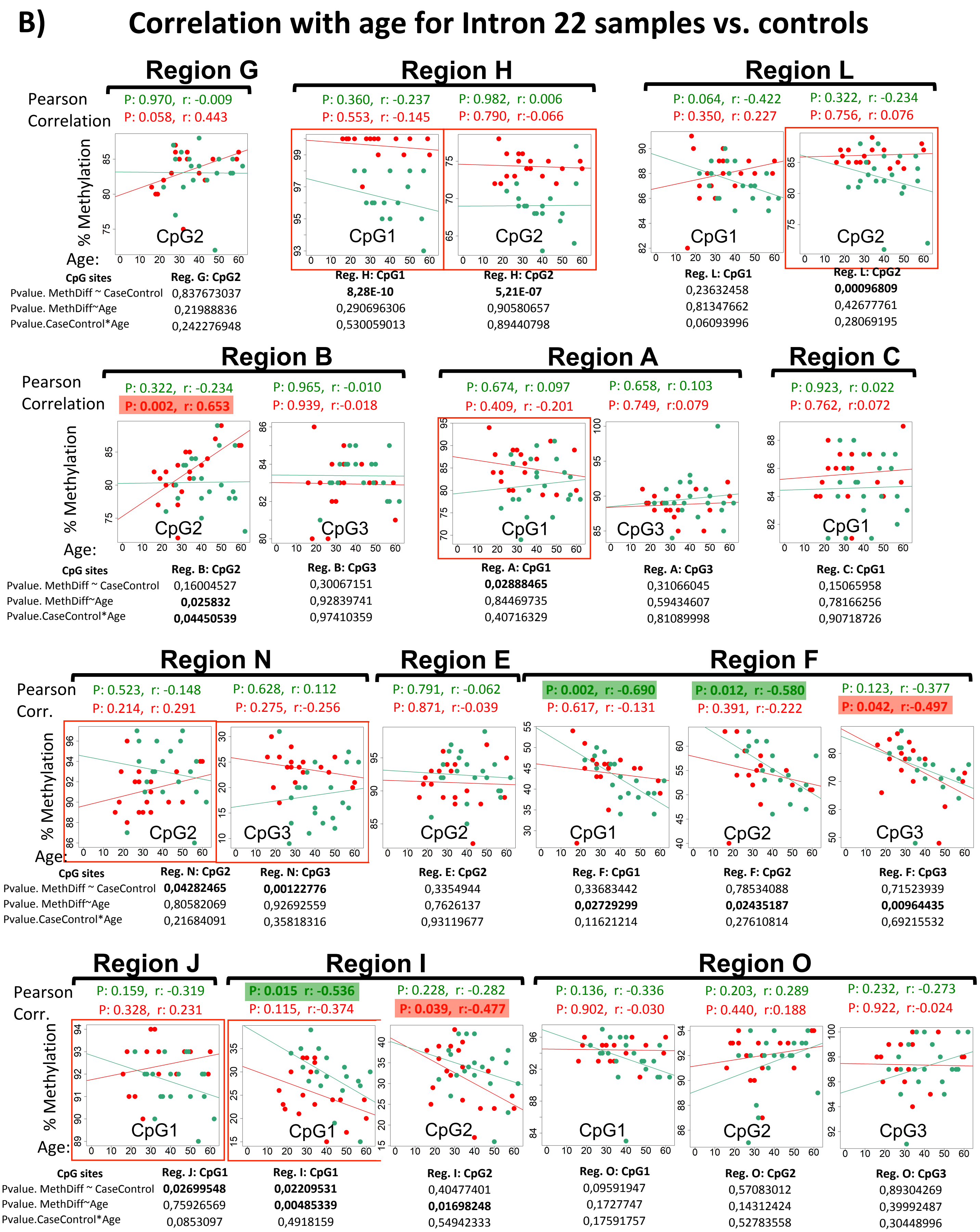

B)
Correlation with age for Intron 22 samples vs. controls
Region G
Pearson Correlation
P: 0.970, r: -0.009
P: 0.058, r: 0.443
% Methylation
Age:
CpG2
CpG sites
Reg. G: CpG2
Pvalue. MethDiff ~ CaseControl 0,837673037
Pvalue. MethDiff~Age 0,21988836
Pvalue.CaseControl*Age 0,242276948
Region H
P: 0.360, r: -0.237
P: 0.553, r: -0.145
P: 0.982, r: 0.006
P: 0.790, r:-0.066
CpG1
CpG2
Reg. H: CpG1 8,28E-10 0,290696306 0,530059013
Reg. H: CpG2 5,21E-07 0,90580657 0,89440798
Region L
P: 0.064, r: -0.422
P: 0.350, r: 0.227
P: 0.322, r: -0.234
P: 0.756, r: 0.076
CpG1
CpG2
Reg. L: CpG1 0,23632458 0,81347662 0,06093996
Reg. L: CpG2 0,00096809 0,42677761 0,28069195
Region B
Pearson Correlation
P: 0.322, r: -0.234
P: 0.002, r: 0.653
P: 0.965, r: -0.010
P: 0.939, r:-0.018
% Methylation
Age:
CpG2
CpG3
CpG sites
Reg. B: CpG2
Pvalue. MethDiff ~ CaseControl 0,16004527
Pvalue. MethDiff~Age 0,025832
Pvalue.CaseControl*Age 0,04450539
Reg. B: CpG3 0,30067151 0,92839741 0,97410359
Region A
P: 0.674, r: 0.097
P: 0.409, r: -0.201
P: 0.658, r: 0.103
P: 0.749, r:0.079
CpG1
CpG3
Reg. A: CpG1 0,02888465 0,84469735 0,40716329
Reg. A: CpG3 0,31066045 0,59434607 0,81089998
Region C
P: 0.923, r: 0.022
P: 0.762, r:0.072
CpG1
Reg. C: CpG1 0,15065958 0,78166256 0,90718726
Region N
Pearson Corr.
P: 0.523, r: -0.148
P: 0.214, r: 0.291
P: 0.628, r: 0.112
P: 0.275, r:-0.256
% Methylation
Age:
CpG2
CpG3
CpG sites
Reg. N: CpG2
Pvalue. MethDiff ~ CaseControl 0,04282465
Pvalue. MethDiff~Age 0,80582069
Pvalue.CaseControl*Age 0,21684091
Reg. N: CpG3 0,00122776 0,92692559 0,35818316
Region E
P: 0.791, r: -0.062
P: 0.871, r: -0.039
CpG2
Reg. E: CpG2 0,3354944 0,7626137 0,93119677
Region F
P: 0.002, r: -0.690
P: 0.617, r: -0.131
P: 0.012, r: -0.580
P: 0.391, r:-0.222
P: 0.123, r: -0.377
P: 0.042, r:-0.497
CpG1
CpG2
CpG3
Reg. F: CpG1 0,33683442 0,02729299 0,11621214
Reg. F: CpG2 0,78534088 0,02435187 0,27610814
Reg. F: CpG3 0,71523939 0,00964435 0,69215532
Region J
Pearson Corr.
P: 0.159, r: -0.319
P: 0.328, r: 0.231
% Methylation
Age:
CpG1
CpG sites
Reg. J: CpG1
Pvalue. MethDiff ~ CaseControl 0,02699548
Pvalue. MethDiff~Age 0,75926569
Pvalue.CaseControl*Age 0,0853097
Region I
P: 0.015 r: -0.536
P: 0.115, r:-0.374
P: 0.228, r: -0.282
P: 0.039, r:-0.477
CpG1
CpG2
Reg. I: CpG1 0,02209531 0,00485339 0,4918159
Reg. I: CpG2 0,40477401 0,01698248 0,54942333
Region O
P: 0.136, r: -0.336
P: 0.902, r: -0.030
P: 0.203, r: 0.289
P: 0.440, r:0.188
P: 0.232, r: -0.273
P: 0.922, r: -0.024
CpG1
CpG2
CpG3
Reg. O: CpG1 0,09591947 0,1727747 0,17591757
Reg. O: CpG2 0,57083012 0,14312424 0,52783558
Reg. O: CpG3 0,89304269 0,39992487 0,30448996
