# Supplementary figures and images for: F8 Inversions at Xq28 Causing Hemophilia A Are Associated With Specific Methylation Changes: Implication for Molecular Epigenetic Diagnosis
Source: Front Genet. 2019 May 29;10:508. doi: 10.3389/fgene.2019.00508 (PMC6548806; doi:10.3389/fgene.2019.00508)

## A) Regression curves: Methyl. vs. Age

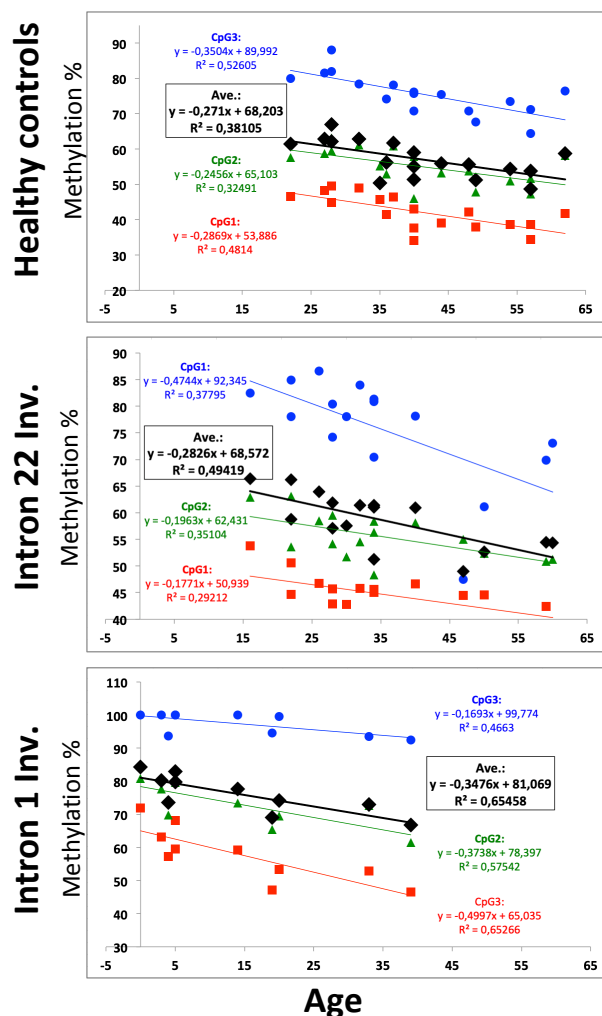

## B) Observed and expected meth. values

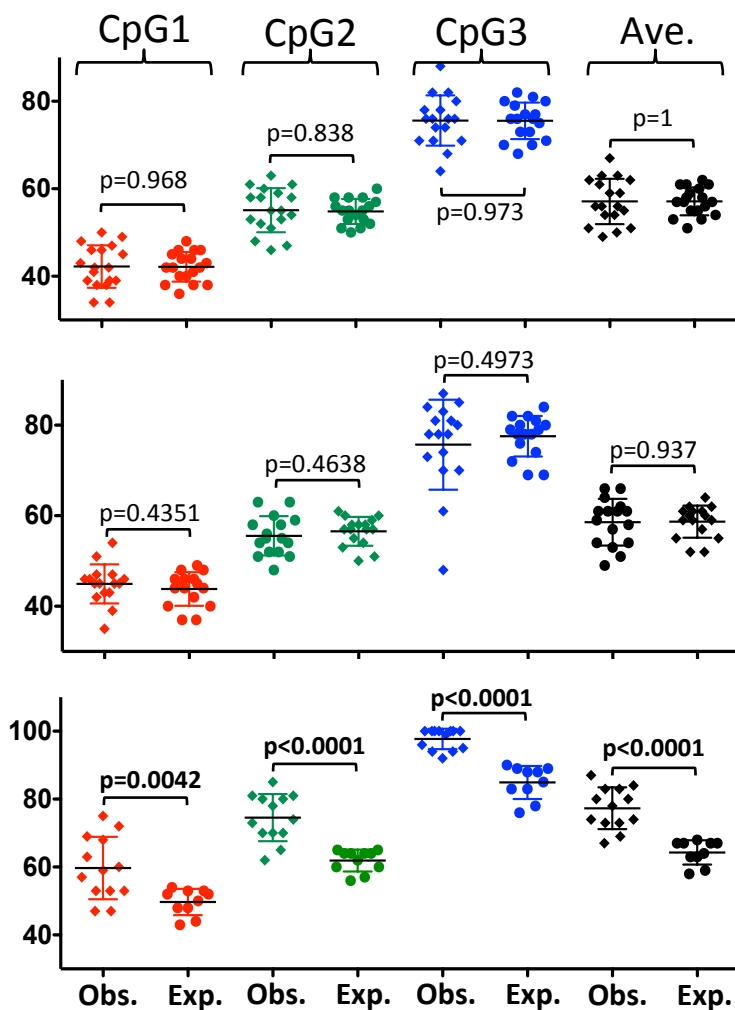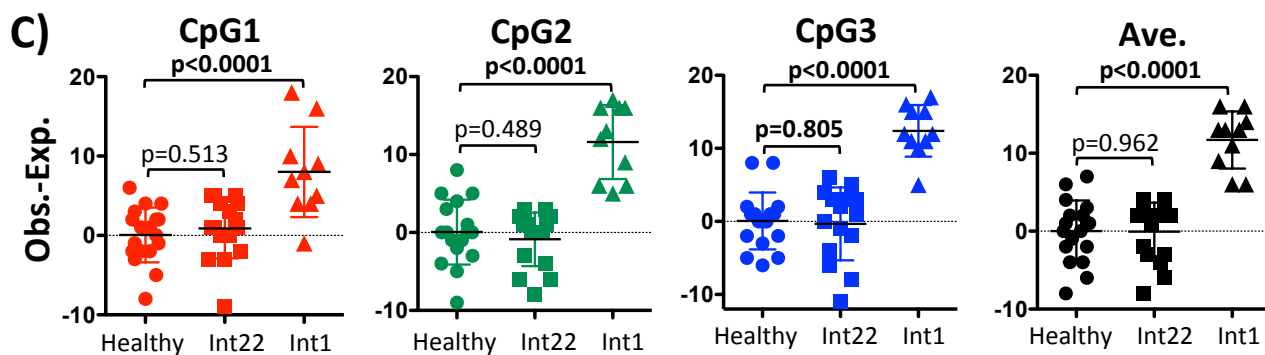

Supplement: FIGURE S3 — Calculation of observed vs. expected methylations values according to predicted linear regression formula of methylation vs. age of healthy group. (A) Linear regression curves of methylation vs. age for the three groups of samples (intron 1-inversion samples, intron 22-inversion samples and healthy controls). Also, equations are shown for every CpG (red, green, and blue for CpGs 1, 2, and 3, respectively) and for the average methylation of three CpGs (in black). (B) Comparison between observed and calculated expected values according to the linear regression equation of healthy controls. T-test p-values showed significance of all CpGs and their average only in the intron 1 inversion group. (C) Comparisons of observed-expected methylation values between inversion groups and healthy controls. [file Data_Sheet_2.PDF]
